# Common Variants of the Liver Fatty Acid Binding Protein Gene Influence the Risk of Type 2 Diabetes and Insulin Resistance in Spanish Population

**DOI:** 10.1371/journal.pone.0031853

**Published:** 2012-03-02

**Authors:** Maria Luisa Mansego, Fernando Martínez, Maria Teresa Martínez-Larrad, Carina Zabena, Gemma Rojo, Sonsoles Morcillo, Federico Soriguer, Juan Carlos Martín-Escudero, Manuel Serrano-Ríos, Josep Redon, Felipe Javier Chaves

**Affiliations:** 1 Genotyping and Genetic Diagnosis Unit, Fundación de Investigación del Hospital Clínico de Valencia-INCLIVA, Valencia, Spain; 2 Fundación de Investigación del Hospital Clínico de Valencia- INCLIVA; Hypertension Clinic, Hospital Clínico Universitario, University of Valencia, Valencia, Spain; 3 Hospital Clínico San Carlos, Department of Internal Medicine II, Plaza Cristo Rey, Madrid, Spain; 4 Endocrinology and Nutrition Department, Carlos Haya University Hospital, Málaga, Spain; 5 Internal Medicine department. Hospital Rio Hortega, University of Valladolid, Valladolid, Spain; 6 Centro de Investigación Biomédica en Red (CIBER) de Diabetes y Enfermedades Metabólicas Asociadas “CIBERDEM”, Institute of Health Carlos III, Ministry of Health, Madrid, Spain; 7 Centro de Investigación Biomédica en Red (CIBER) de Fisiopatología, Obesidad y Nutrición [CIBEROB (CIBER 03/06)], Institute of Health Carlos III, Ministry of Health, Madrid, Spain; 8 Centro de Investigación Biomédica en Red (CIBER) de Diabetes y Enfermedades Metabólicas Asociadas (CIBERDEM), Institute of Health Carlos III, Ministry of Health, Madrid, Spain; John Hopkins Bloomberg School of Public Health, United States of America

## Abstract

**Summary:**

The main objective was to evaluate the association between SNPs and haplotypes of the *FABP1-4* genes and type 2 diabetes, as well as its interaction with fat intake, in one general Spanish population. The association was replicated in a second population in which HOMA index was also evaluated.

**Methods:**

1217 unrelated individuals were selected from a population-based study [Hortega study: 605 women; mean age 54 y; 7.8% with type 2 diabetes]. The replication population included 805 subjects from Segovia, a neighboring region of Spain (446 females; mean age 52 y; 10.3% with type 2 diabetes). DM2 mellitus was defined in a similar way in both studies. Fifteen SNPs previously associated with metabolic traits or with potential influence in the gene expression within the *FABP1-4* genes were genotyped with SNPlex and tested. Age, sex and BMI were used as covariates in the logistic regression model.

**Results:**

One polymorphism (*rs2197076*) and two haplotypes of the *FABP-1* showed a strong association with the risk of DM2 in the original population. This association was further confirmed in the second population as well as in the pooled sample. None of the other analyzed variants in *FABP2*, *FABP3* and *FABP4* genes were associated. There was not a formal interaction between *rs2197076* and fat intake. A significant association between the *rs2197076* and the haplotypes of the *FABP1* and HOMA-IR was also present in the replication population.

**Conclusions:**

The study supports the role of common variants of the *FABP-1* gene in the development of type 2 diabetes in Caucasians.

## Introduction

Type 2 diabetes (DM2), obesity and abdominal fat distribution are cardiometabolic risk factors that cluster frequently. In their origin, fetal, environmental and genetic factors may play a role. The genetic susceptibility follows a complex trait inheritance, where the interaction between multiple genes and environmental factors results in the final phenotype. Among the candidate genes, the fatty acid binding protein gene family (*FABP*s) has been studied due to its role in the uptake, intracellular metabolism and excretion of long chain fatty acids (LCFA) [1]. Several authors linked polymorphisms of these genes with different metabolic phenotypes. Two missense mutations, the *c.163 A>G* (*p.Ala54Thr*) polymorphism of the intestinal *FABP* (*FABP2*) [2], , the *c.280 A>G* (*p.Thr94Ala*) of the liver *FABP* (*FABP1*) [6], [7] and one polymorphism in the promoter region of the adipose *FABP* (*FABP4*) [8] were frequently related with metabolic abnormalities including DM2. In addition, SNPs of the muscular *FABP* (*FABP3*) have been linked to DM2 in an Asian population [9]. Furthermore, polymorphisms of this gene family have also been linked to cardiovascular risk and coronary heart disease [10]. The potential interaction of these genes with fat dietary content has not been clearly elucidated but for a few papers [5].

The purpose of the present study was to evaluate the association between specific SNPs and haplotypes of the *FABP1*-*4* genes and DM2, obesity and abdominal obesity in two independent populations. Besides, we evaluated the potential interaction of these genetic variants with total, saturated and polyunsaturated fat intake as well as the genetic association with Homeostasis Assessment Index (HOMA-IR) in the replication population.

## Methods

### Study populations

The association analysis was first made in one thousand two hundred and seventeen subjects with high genotyping call rate, selected from a large epidemiologic study which was carried out in the Rio Hortega Hospital of Valladolid [11], [12], [13] (Hortega population). Population selection and methodology have been previously described [12]. In summary, the sample included individuals older than 18 years in the absence of serious concomitant disease or psychiatric disorder, which could interfere with the study. To be representative of the general population, investigators calculated the sample size by using local public resources and finally 1502 subjects were included. For replication, we selected eight hundred and five subjects from another population-based epidemiological study, in Segovia, which is also located in a central region of Spain. This study also included a large number of subjects but in the same way as we did with Hortega, those subjects with low genotyping call rate were not included. Description of the sampling methodology and characteristics of this study were previously published [14], [15].

All the subjects included in the study were Caucasians living in an area with low immigration rate. The local Ethical Committees of the following hospitals: Rio Hortega Hospital from Valladolid and San Carlos Clinical Hospital from Madrid approved the Hortega and the Segovia-VIVA studies respectively and all the participants gave written agreement to participate. Additionally the participants gave their informed consent to use their blood samples for genetic studies.

### Assessment of cardiometabolic risk factors

The two studies included the determination, among others, of anthropometric measurements, blood pressure, glycaemia, lipid profile and smoking status as well as personal and familial information about cardiovascular risk factors.

Body mass index (BMI) was calculated using the following formula “weight (kg)/height (m^2^)”; Weight was assessed with precise scales while the individuals were without shoes and with light clothing. Height was determined in a similar way. A subject was classified as obese if his/her BMI was ≥30 Kg/m^2^. Waist circumference was measured according to standard recommendations. Patients were classified to have central obesity when their waist was >102 and >88 cm for men and women, respectively according to the ATP-III criteria [16].

Assessment of glucose and lipid profile differed in the two study populations. In the Hortega, blood samples were obtained with a mean of 3 hours fasting (range 0–17). Basic serum biochemistry and lipid profile (total cholesterol, HDL cholesterol and triglycerides) were measured with an Hitachi 917 autoanalyzer (Boehringer, Germany). Glucose was measured by the glucose oxidase method and LDL cholesterol was calculated using the Friedewald formula. In Segovia population, blood samples were obtained after 10 hours fasting. Glucose was measured twice by the glucose oxidase method with an autoanalyzer (Hitachi 704, Boehringer Mannheim, Germany). Total cholesterol, triglycerides and HDLc were assessed with an enzymatic method by means of standard analyzers (Boehringer Mannheim, Germany). LDL cholesterol was calculated with the Friedewald formula.

The definition of type 2 diabetes was slightly different in both studies. In Hortega subjects were considered as diabetics if they were already diagnosed of type 2 diabetes by a physician or if the plasma glucose remained equal or higher than 126 mg/dl after the extraction of a second sample in fasting conditions in those subjects with glucose equal or higher than 140 mg/dl in non fasting conditions [11]. By contrast, in Segovia, individuals with fasting glucose equal to or higher than 126 mg/dl, already diagnosed or having antidiabetic treatment were classified as diabetics. In this last population an oral glucose tolerance test was performed and interpreted according to the recommendations of the expert panel for the diagnosis and classification of DM2 [17].

Moreover, in the Segovia population, HOMA-IR index was calculated: fasting insulin (µU/ml) × fasting glucose (mmol/l)/22.5. This information was not available in Hortega because the main objective of this study was to estimate the prevalence of the main cardiovascular risk factors at a population level whereas the aim of the Segovia study was to investigate the prevalence of anthropometrics and physiological parameters related to obesity and others components of the metabolic syndrome.

### Nutrient Intake Assessment

Diet was assessed within the Hortega study by a validated questionnaire, which used two 24-hour recalls and one semi quantitative survey. The nutrient composition was determined with the food composition tables of the nutritional department of the University of Granada and the data were analyzed with the statistical program “BitASDE” and with the license of the Medical School. Total, saturated and polyunsaturated fat intakes were estimated in g/day and their potential interaction with variants of the *FABP* genes was considered.

### Genotyping

DNA was isolated from peripheral blood cells using Chemagic System (Chemagen) and samples were diluted to a final concentration of 100 ng/µl. The DNA samples used in this project were related to the Clinical Hospital Biobank of Valencia.

The genotyping was assessed using an oligo-ligation assay, SNPlex (Applied Biosystems, Foster City, CA). Fifteen SNPs of the *FABP1-4* genes were selected. We included variants with potential influence in the gene and protein function as well as the most important variants described in the literature. Reference names and characteristics of the selected single nucleotide polymorphisms (SNPs) are shown in Table 1.

**Table 1:** Information of the selected polymorphisms for each FABP gene.

Locus	Gene Name	HGN	SNP^1^	CHR position	Reference^2^	Consequence
2p11	Fatty acid-binding protein, liver	*FABP1*	*rs2197076* *	88203873	*c.334-135 G>A*	3 prime UTR
			*rs2241883* *	88205181	*c.280 A>G (p.T94A)*	Non synonymous coding
			rs2970901*	88209949	*c.-1238 G>T*	Upstream
4q28-q31	Fatty acid binding protein 2, intestinal	*FABP2*	*rs1511025*	120459686	*c.357 A>G (p.V119V)*	Synonymous coding
			*rs4834770*	120461297	*c. 216 T>C (p.N72N)*	Synonymous coding
			*rs1799883*	120461344	*c.163 A>G (p.A54T)*	Non synonymous coding
			*rs6857641*	120462959	*c.-254 T>C*	5 prime UTR
			*rs2282688*	120463170	*c.-465 A>G*	Upstream
			*rs10034579*	120463477	*c.-772 A>C*	Upstream
1p33-p32	Fatty acid binding protein 3, muscle and heart	*FABP3*	*rs2271072*	31613013	*c.247-85 C>G*	Intronic
			*−345CT*	31618855	*c.-345 C>T*	Promoter
			*rs12401792* *	31620006	*c. -1559 G>T*	Within non coding gene
8q21	Fatty acid binding protein 4, adipocyte	*FABP4*	*rs8192688* *	82555404	*c.74-16 C>T*	Intronic
			*rs16909225*	82558914	*c.-957 A>G*	Upstream
			*rs2279885*	31845686	*c.73+103G>C*	Intronic

1 , dbSNP 126.

2 , It begins in the first nucleotide of exon 1, Build 126, Ensembl release 41.

HGN: The HUGO Gene Nomenclature.

* Tag-SNP by Hapmap in caucasic subjects.

Genotypes were obtained with the software GeneMapper v4.0 (Applied Biosystems, Foster City, CA). Allelic and genotypic frequencies were determined for every SNP.

We used the following filters in this study: 90% as the lowest call rate for individuals and SNPs, 1% as the lowest minor allele frequency for SNPs and 0.001 as p-value for Hardy-Weinberg equilibrium.

### Statistical analysis

All values were expressed as mean ± standard deviation. ANOVA test was used to compare quantitative variables between groups and chi-square test for categorical variables using the statistical software StataIC 11 (StataCorp4905 Lakeway drive ,College Station, Texas, 77845, USA).

Genetic association analysis with type 2 diabetes, obesity (based on the BMI values) and abdominal obesity (based on the waist circumference values) were sought by logistic regression models adjusted for age and sex in the case of obesity and also for BMI in the case of abdominal obesity and type 2 diabetes. The interaction with fat intake was assessed by including a multiplicative term within the adjusted logistic regression models. Total, saturated, monounsaturated and polyunsaturated fat intakes were included in the model as quantitative traits but also as qualitative traits based on quartiles and tertiles of the continuous variable. Logistic and linear regression models adjusted by age, sex and BMI were used to evaluate the association of the selected SNPs with insulin resistance expressed as a dichotomous trait or as a continuous trait in Segovia population. The cut-off to consider insulin resistance was 3.8 in agreement with the recommendations of Ascaso et al(18) for Spanish populations. This association was performed with or without including those subjects with type 2 diabetes.

Linkage disequilibrium (LD) was estimated by R-square and haplotype frequencies by the Expectation Maximization Algorithm (EM) [18]. Haplotype association was analyzed by logistic regression adjusted by the same covariables as the individual SNP analysis and using the sliding window approach up to six markers and a proxy approach taking into account the results of the individual SNP association. Tag-SNPs, LD and haploblocks were calculated using Haploview version 3.32 [19].

We used the free software Quanto v1.2.4 [20], [21] to calculate the statistical power with the qualitative traits under additive and dominant models taking into account the minor allele frequency of the selected SNPs, the final sample size, the prevalence of the metabolic traits in our sample and different genotypes relative risk. For the majority of the variants we had more than 80% of power to detect associations with an effect size of 1.8 or higher and with a type I error of 0.05. The individual SNP and haplotype analysis was performed with the program PLINK v.1.06 developed by Purcell (http://pngu.mgh.harvard.edu/purcell/plink/). To deal with the multiple comparisons problem we used the Bonferroni p-value to consider a result as significant.

## Results

Although the genotyping procedure was performed in 1502 subjects from the Hortega study and 1241 subjects from the Segovia study, after excluding those subjects with call rate lower than 90% (285 subjects in Hortega and 436 in Segovia), the final sample sizes were 1217 in Hortega and 805 in Segovia. The genotyping call rate for the remaining individuals was 98.8 in Hortega and 98.6% in Segovia. The individuals from the latter population were younger (52 y vs 54 y), had higher BMI, total cholesterol, and lower levels of triglycerides and systolic blood pressure than individuals from the Hortega study had. The prevalence of DM2, obesity and abdominal obesity was higher in Segovia than in Hortega population. Only 84 (6.9%) out of 1217 subjects in Hortega were under hypolipemiant treatment. In Segovia, this information was missing in a great proportion of patients. From the 210 patients in whom the information about the lipid lowering treatment was available, 54 (25.7%) were under hypolipemiant treatment. The main characteristics of the selected individuals from both populations are shown in Table 2.

**Table 2:** General characteristics of the studied subjects.

Characteristic	Pooled population	Hortega	Segovia-Viva
No. of Subjects	2022	1217	805
Age (y)	54±16.6	54.8±19.3	52.8±11.2**
Sex (Male/female)	971/1051	612/605	359/446*
Body Mass Index (kg/m^2^)	26.8±4.2	26.4±4.2	27.5±4.1***
Waist Perimeter (cm)	91.1±12.3	89.4 ±12.9	93.7±10.8***
Obesity (n, %)	408 (20.7)	206 (17.6)	202(25.2)***
Abdominal Obesity (n, %)	720 (36.5)	357 (30.4)	363 (45.4)***
Diabetes Mellitus (n, %)	174 (8.9)	95 (7.8)	79 (10.34)^0.052^
Plasma Glucose (mg/dL)	91.6±19.6	92.1±19.4	90.9±19.8
HOMA INDEX	NA	NA	2.97±2.6
Hypertension (n, %)	851(42.3)	510 (41.9)	341 (43.0)
Systolic Blood Pressure (mmHg)	128.4±20.3	130.7±21.3	124.8±18.1***
Diastolic Blood Pressure (mmHg)	78.9±10.4	79.3±10.6	78.3±10.1*
Total Cholesterol (mg/dL)	206.6±39.1	201.0±38.3	215.7±38.7***
HDL-Cholesterol (mg/dL)	54.1±15.7	51.8±14.1	57.7±17.3***
Triglycerides (mg/dL)	147.7±104.8	175.5±109.9	103.2±77.7***
Lipid lowering treatment (n, %)	138 (9.6)^1^	84 (6.9)	54 (25.7)^2^
Total fat intake:	NA	97.6±37.8	NA
Monounsaturated	NA	40.1±17.6	NA
Polyunsaturated	NA	13.6±6.9	NA
Saturated	NA	28.7±12.0	NA

*Values are mean ± standard deviation or absolute number (percentage); NA: Not available.*

* *Significant differences between the two populations p-value<0.05.*

** *Significant differences between the two populations, p-value<0.01.*

*** *Significant differences between the two populations, p-value<0.001.*

1 *This information was available for 1427 subjects.*

2 *This information was available for 210 subjects.*

In Hortega population the SNP, *rs1799883*, within the *FABP2* gene was excluded because of low genotyping call rate. The other fourteen SNPs passed the pre-defined thresholds and the call rate for the remaining SNPs was 98.8%. In Segovia population, two SNPs (*rs2970901* of FABP1 and *rs2279885* of FABP3) were excluded because of low genotyping call rate or MAF lower than 1%. The other thirteen SNPs passed the pre-defined thresholds and the call rate for the remaining SNPs was 98.6%. In the pooled analysis, three SNPs (*rs4834770 and rs1799883 on FABP2* and *rs2279885* on *FABP3*) were excluded. The other twelve SNPs passed the pre-defined thresholds and the call rate for the remaining SNPs was 98.5%. The SNP call rates for each population and for the pooled sample after exclusion of subjects and SNPs with low genotyping call rate are shown in Table S1.

### Individual SNPs association with type 2 diabetes, obesity and abdominal obesity

One polymorphism of the *FABP1* gene was strongly associated with the risk of type 2 diabetes in Hortega population even when Bonferroni test was applied for multiple comparisons. The allele *A* of the *rs2197076* increases the risk of having DM2 under additive but especially under dominant model [OR 2.15 (1.36–3.40) p-value 0.001004; Bonferroni p-value 0.0140]. This result was further confirmed in Segovia population [OR 2.05 (1.25–3.36) for the dominant model, p-value 0.0041; Bonferroni p-value 0.053] and in the pooled analysis [OR 2.10 (1.50–2.92) for the dominant model, p-value 1.31E-05; Bonferroni p-value 0.00015].

The association among variants of the other FABP genes and the other metabolic traits, obesity and abdominal obesity, was quite inconclusive (Tables S2 and S3). The individual SNP association analyses for the additive, dominant and recessive models are shown in Table 3.

**Table 3:** Individual SNPs and haplotypes association with type 2 diabetes.

					Hortega		Segovia		Pooled	
Individual SNP association adjusted by age, sex and BMI										
SNP	CHR	A1		TEST	OR(95% CI)	p-value	OR(95% CI)	p-value	OR(95% CI)	p-value
rs2197076	2	A		ADD	1.90 (1.28–2.82)*	0.001472 (0.0206)**	1.75 (1.16–2.63)*	0.007461 (0.097)**	1.83 (1.38–2.43)*	2.86E-05 (0.00034)**
				DOM	2.15 (1.36–3.40)*	0.001004 (0.01406)**	2.05 (1.25–3.36)*	0.0041 (0.053)**	2.10 (1.50–2.92)*	1.31E-05 (0.00015)**
				REC	1.68 (0.46–6.19)*	0.4356 (1)**	1.44 (0.40–5.18)*	0.5757 (1)**	1.61 (0.65–4.00)*	0.300 (1)**

Haplotype association adjusted by age, sex and BMI (sliding window approach)												
NSNP	CHR	SNP1	SNP2	HAP	Freq (H/S) ***		OR	p-value	OR	p-value	OR	p-value
2	2	rs2197076	rs2241883	AT	0.174/0.164		1.81	0.0039	1.76	0.00692	1.79	7.5E-05
2	2	rs2197076	rs2241883	GT	0.482/0.47		0.69	0.0276	0.59	0.00432	0.696	0.00277

Haplotype association adjusted by age, sex and BMI (proxy approach)												
NSNP	CHR	SNP1	SNP2	HAP	Freq (H/S) ***		OR	p-value	OR	p-value	OR	p-value
2	2	rs2197076	rs2241883	AT	0.174/0.168		1.61	0.00762	1.83	0.00244	1.69	7.6E-05
2	2	rs2197076	rs2241883	GT	0.482/0.47		0.754	0.07	0.58	0.00151	0.67	0.00056

*CHR: chromosome; A1: minor allele; OR: odds ratio;*

* *Confidence interval for the OR;*

** *p-value after Bonferroni correction:*

*** *Haplotype frequency in Hortega and Segovia.*

*ADD: additive model; DOM: dominant; REC: recessive.*

The total counts and frequencies of the different alleles and genotypes of the selected SNPs according to the diabetes state in each population have been summarized in Table S4.

### Haplotype association analysis with type 2 diabetes, obesity and abdominal obesity

**Figure 1:**
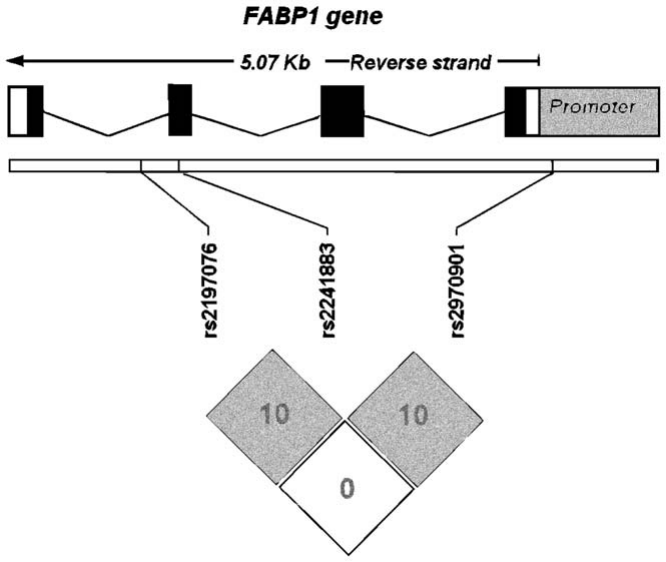
Linkage disequilibrium of the associated region of FABP1 using the R-square color scheme.

One haplotype including the allele *A* of the *rs2197076* and the allele *T* of the *rs2241883* (*p.Thr94Ala*) significantly increased the risk of having type 2 diabetes in Hortega, Segovia and in the pooled analysis [OR 1.81 in Hortega, p-value 0.0039; OR 1.76 in Segovia, p-value 0.00692; OR 1.79 in the pooled analysis, p-value 7.5E-05]. Another haplotype including the *G* allele of the *rs2197076* resulted in protection against type 2 diabetes, the results being highly significant in Segovia and in the pooled analysis. The proxy approach also found significant association with both the individual marker and the haplotype, which reinforces the results. Figure 1 shows the LD of that region of chromosome 2 with the two markers (R-sq<0.8).

### SNP and haplotype association with HOMA-IR

We evaluated the possible association of polymorphisms of the *FABP-1* gene with insulin resistance in the replication population. There was a significant association between genotypes of the *rs2197076* polymorphism and HOMA-IR values expressed as a quantitative trait in the dominant model. Carriers of the allele *A* of this SNP had HOMA-IR values around 0.60 units higher than homozygotes *GG*. The results were significant even when the Bonferroni correction was applied but this association weakened after exclusion of diabetics subjects under treatment and completely disappeared after exclusion of subjects with type 2 diabetes.

Regarding the haplotype association analysis, the previously associated haplotypes with type 2 diabetes were also significantly associated with HOMA-IR expressed as a quantitative trait but as it happened with the individual analysis, there was a weaker association after removing those subjects under diabetic treatment and there was no association after exclusion of all the type 2 diabetic subjects.

**Figure 2:**
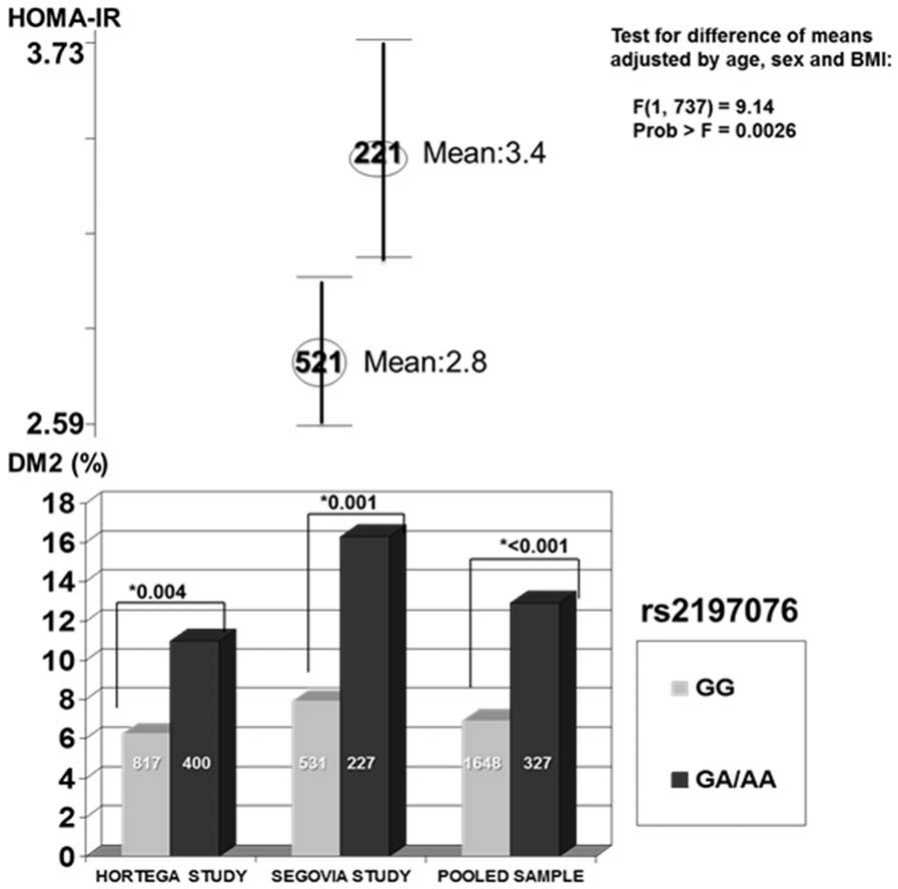
Prevalence of type 2 diabetes and HOMA-IR values according to genotypes of the *rs2197076*. [Bottom: Prevalence of DM2 for genotypes of the rs2197076 under dominant model. Inside the bars are shown the number of individuals for each genotype; Top: Mean of HOMA-IR for genotypes of the rs2197076 in Segovia population under dominant model. Inside the circles are the number of individuals for each genotype. *Chi-square test for the comparison of type 2 diabetes prevalence between genotypes].

The individual SNP and haplotype association analyses with HOMA-IR for all subjects, all except diabetics under treatment and exclusively for non diabetic individuals are shown in Table 4. The prevalence of type 2 diabetes according to genotypes of the *rs2197076* under dominant model in Hortega, Segovia and in the pooled analysis is shown in Figure 2 as well as the HOMA-IR values in the Segovia study.

**Table 4:** Individual SNPs and haplotypes association with HOMA index in Segovia population.

					HOMA index >3.8 (YES/NO)		HOMA Index (continuous trait)		Group: ALL(153 CASES/598 CONTROLS); ALL^¶^ (142 CASES/591 CONTROLS); EXCLUDING DIABETICS (108 CASES/568CONTROLS)
Individual SNP association adjusted by age, sex and BMI									
SNP	CHR	A1		TEST	OR	p-value	Beta	p-value	
rs2197076	2	A		ADD	1.19(0.85–1.68)*	0.3035 (1)**	0.4614(0.126–0.796)*	0.007156 (0.09303)**	ALL
					1.18(0.83–1.68)*	0.338 (1)**	0.429(0.09–0.76)*	0.0127 (0.165)**	ALL^¶^
					1.14(0.66–1.69)*	0.523(1)**	0.22(−0.06–0.51)*	0.1287(1)**	EXCLUDING DIABETICS
rs2197076	2	A		DOM	1.25(0.83–1.86)*	0.2763 (1)**	0.604(0.212–0.996) *	0.002588 (0.03365)**	ALL
					1.22(0.81–1.85)*	0.329 (1)**	0.558(0.163–0.953) *	0.005707 (0.0742)**	ALL^¶^
					1.14(0.71–1.82)*	0.5762(1)**	0.27(−0.06–0.61)*	0.111(1)**	EXCLUDING DIABETICS
rs2197076	2	A		REC	1.16(0.40–3.34)*	0.7835 (1)**	0.1831(−0.834–1.2)*	0.7243(1)**	ALL
					1.21(0.42–3.49)*	0.716 (1)**	0.2145(−0.797–1.22)*	0.6781(1)**	ALL^¶^
					1.33(0.41–4.29)*	0.6267(1)**	0.21(−0.6–1.1)*	0.6266(1)**	EXCLUDING DIABETICS

Haplotype association adjusted by age, sex and BMI (sliding window approach))											
NSNP	CHR	SNP1	SNP2	HAP	Freq***		OR	p-value	Beta	p-value	
2	2	rs2197076	rs2241883	AT	0.163		1.25	0.177	0.533	0.00517	ALL
					0.162		1.26	0.185	0.509	0.005	ALL^¶^
					0.153		1.20	0.357	0.271	0.0794	EXCLUDING DIABETICS
2	2	rs2197076	rs2241883	GT	0.47		0.813	0.106	−0.208	0.128	ALL
					0.48		0.845	0.197	−0.162	0.24	ALL^¶^
					0.489		0.894	0.442	−0.0354	0.76	EXCLUDING DIABETICS

ALL^¶^ does not included those 31 diabetics under treatment; *CHR: chromosome; A1: minor allele; OR: odds ratio;*

* *Confidence interval for the OR or beta parameter;*

** *p-value after Bonferroni correction:*

*** *Haplotype frequency; ADD: additive model; DOM: dominant, REC: recessive.*

### Genetic and environment interaction

In Hortega population, the strength of association between genotypes and haplotypes of the *FABP1* and DM2 were slightly higher in the group of low fat intake [OR 2.56, p-value 0.0056] than that observed in the group of high fat intake [OR 2.04, p-value 0.027]. No formal interaction among fat intake and genotypes of the *rs2197076* for the risk of type 2 diabetes was found. Saturated or poly-unsaturated fat intake, expressed as a quantitative trait or as a qualitative trait, did not interact with the genotypes in the risk of type 2 diabetes. The individual SNP and haplotype association analysis according to the groups of fat intake are shown in Table 5.

**Table 5:** Individual SNPs and haplotypes association with type 2 diabetes according to the level of fat intake in Hortega population.

					LOW FAT INTAKE [individuals in the first and second quartiles of total fat intake(37 cases/431 controls)]		HIGH FAT INTAKE [individuals in the third and fourth quartiles of total fat intake (37 cases/434 controls)]	
Individual SNP association adjusted by age, sex and BMI								
SNP	CHR	A1		TEST	OR	Bonferroni p-value	OR	Bonferroni p-value
rs2197076	2	A		ADD	1.951 (1.08–4.06)*	0.04202	1.851(1.03–3.84)*	0.03274
rs2197076	2	A		DOM	2.564 (1.04–3.84)*	0.005676	2.045 (0.94–3.44)*	0.02715
rs2197076	2	A		REC	0.8829 (0.09–6.68)*	0.9091	1.507 (0.84–2.66)*	0.7099

Haplotype association adjusted by age, sex and BMI										
NSNP	CHR	SNP1	SNP2	HAP	Freq***		OR	p-value	OR	p-value
2	2	rs2197076	rs2241883	AT	0.174/0.178		2.03	0.0335	1.8	0.0791
2	2	rs2197076	rs2241883	GT	0.461/0.487		0.688	0.156	0.847	0.521

*CHR: chromosome; A1: minor allele; OR: odds ratio;*

* *Confidence interval for the OR:*

*** *Haplotype frequency for low and high intake groups.*

*ADD: additive model; DOM: dominant; REC: recessive.*

## Discussion

The data from the present study expand previous studies of association of *FABP* genes in the risk of having type 2 diabetes and insulin resistance. One single nucleotide polymorphism of the *FABP-1* gene, *rs2197076* and one haplotype were associated with an increased risk of type 2 diabetes even when adjusted for age, sex and BMI. The replication analysis, not only for the individual SNP but also for the haplotype in a population with a different metabolic profile supports our initial findings. In this last population, the SNP and the haplotype could also be related with HOMA-IR. Carriers of the *A* allele of the *rs2197076* of *FABP1* or those with the haplotype *AT* of the *rs2197076* and *rs2241883* had significantly higher levels of HOMA-IR than those with the major allele *G* of the *rs2197076*.

The evaluation of the total, saturated or poly-unsaturated fat intake, as potential environmental factors, which could influence the genetic association, was inconclusive. Although the level of association was more evident in the low fat intake group, the formal test for interaction was not significant.

The implication of the *FABP* genes on the risk of DM2 or insulin resistance has been supported by experimental studies in animals as well as in humans [22]. Liver fatty acid binding protein (*FABP1*) is an abundant cytosolic lipid-binding protein that regulates lipid transport and metabolism. Deletion of the *FABP1* gene shows no obvious phenotype in mice receiving a low fat chow diet, but leads to decreased hepatic triglyceride accumulation in the prolonged fasted state, which exposes mice to an increased fatty acid flux to the liver. The function of the *FABP1* gene may be regulated by polymorphisms in coding regions, probably leading to modifications in hepatic triglycerides accumulation and hepatic insulin resistance. Impairment of the function of this gene can lead to several metabolic disturbances such as insulin resistance, non-alcoholic esteatohepatitis and several components of the so-called metabolic syndrome [23].

Among all the polymorphisms which have been tested for their relationship with obesity and type 2 diabetes within the *FABP1* gene, the functional mutation *rs2241883* (*p.Thr94Ala*) has been the most studied. According to our results while there was no association with the *p.Thr94Ala* in our populations, haplotypes containing the allele *T* (wild allele) were significantly associated with the risk of type 2 diabetes in both populations and in the pooled analysis. The *p.Thr94Ala* polymorphism induces an amino-acid change that is located within the N-terminal region of the protein which is a component of the fatty acid binding site and therefore the binding capacity might be reduced in patients with the *94Ala* variant [24]. In the study carried out by Brouillette et al, they found that carriers of the *94Ala* allele had higher baseline FFA, lower BMI and waist circumference than homozygotes *Thr94Thr* [6]. In our associated haplotypes, the direction of the association was driven by alleles of the *rs2197076* and not by alleles of the functional mutation. Our results suggest that the most strongly associated SNP (*rs2197076*), because of its location in the 3 prime UTR site and its association with a potential functional variant in one risk haplotype, could regulate the functional activity of the protein individually or in haplotypes. It is likely that the *A* allele of the *rs2197076* might therefore reduce *FABP1* gene expression thereby contributing to enhance that situation of IR-lipotoxicity, IR and DM2.

Type 2 diabetes and insulin resistance are intimately related and therefore it is very difficult to separate one from the other. The association with the HOMA-IR index values disappeared after the exclusion of diabetic subjects what supports the association with type 2 diabetes. We cannot be sure if the association with type 2 diabetes is mediated through insulin resistance or not. It is well-known that both traits, insulin resistance and type 2 diabetes are strongly influenced by genetics with an estimated heritability for insulin resistance of around 60% and around 35–54% for the plasma insulin levels in familial and twin studies [25]. Type 2 diabetes patients usually have lipid abnormalities with high levels of plasma triglycerides and free fatty acids eventually leading to increased FFA flow from the peripheral tissues (muscle, liver) causing lipotoxicity and hence IR.

We did not find an association of *FABP1* variants with other diabetes related traits such as hypertriglyceridemia, hypertension, low HDL or high LDL cholesterol. Only there was a trend for the association with hypertriglyceridemia and hypercholesterolemia LDL but only in Segovia population and only with the qualitative trait but not with the quantitative trait. The same occurred for several variants of the intestinal *FABP* gene which were close to the significance after Bonferroni correction with hypertriglyceridemia but only in Segovia and only with the qualitative trait.

The main limitations of the present study are the lack of FFA levels in our populations and the absence of functional studies of the gene variants. Other limitation was related to the low genotyping call rate observed in many of the samples (around 19% in Hortega and 35% in Segovia). We decided to exclude those samples in an attempt to not compromise the results. We believe that the problems with those subjects with very low call rate were due to DNA problems although the amount of DNA and the ratio 260/280 were within the optimal range. After the exclusion of these problematic samples the genotyping call rate was very high as it has to be because of the high accuracy of the SNPlex platform [26]. The major strength is that we were able to replicate the results in an independent Spanish population. Since the two populations belong to the same geographical region and that region has a low immigration rate, we do not expect bias in our results due to population stratification. For this reason as the genetic background was expected to be the same in both studies we decided the pooled strategy over other strategies such as meta-analysis in order to increase our sample size what is crucial to detect variants of low risk. The different metabolic profile observed between populations could be related with the different sampling methodology but especially with the different characteristics of the target populations. Individuals of Segovia population were selected in a primary care environment and the majority belonged to rural areas. They were also significantly younger than those from the Hortega study. The individuals from the latter study were recruited in the area covered by a tertiary hospital and majority of them lived in urban areas. This population was also even regarding to gender distribution compared with Segovia population which included mostly females. Because of the potential influence of the population to which each individual belongs, we also adjusted the analysis by this factor, and the association of the polymorphism *rs2197076* and type 2 diabetes remained highly significant [OR 1.83 (1.38–2.43), Bonferroni p-value 0.0003 for the additive model and OR 2.11 (1.51–2.95), Bonferroni p-value 0.00013 for the dominant model] .

Also important is the inclusion of fat intake as a possible factor of interaction between the gene variants and metabolic traits.

In summary, our study supports the role of the liver *FABP* in the development of type 2 diabetes and insulin resistance in representative samples of Spanish general population. Functional studies may clarify in the future if the liver *FABP* may or may not be a potential target for treatment of type 2 diabetes and insulin resistance.

## Supporting Information

Table S1 **SNP call rates for each population and for the pooled sample after exclusion of subjects and SNPs with low genotyping call rate.**
(DOCX)

Table S2 **Association among genotypes of the** ***rs2197076*** **and diabetes related traits under an additive inheritance genetic model.**
(DOCX)

Table S3 **Association among polymorphisms of the** ***FABP2*** **gene and hypertriglyceridemia under an additive inheritance genetic model.**
(DOCX)

Table S4 **Alleles and genotypes frequencies for the analyzed polymorphism in the pooled sample, Hortega and Segovia population separated by type 2 diabetes status.**
(DOCX)
