# Supplementary material for: Common Variants of the Liver Fatty Acid Binding Protein Gene Influence the Risk of Type 2 Diabetes and Insulin Resistance in Spanish Population
Source: PLoS One. 2012 Mar 2;7(3):e31853. doi: 10.1371/journal.pone.0031853 (PMC3292554; doi:10.1371/journal.pone.0031853)
Supplement: Table S1 — SNP call rates for each population and for the pooled sample after exclusion of subjects and SNPs with low genotyping call rate. (DOCX) [file pone.0031853.s001.docx]

|  |  | **POOLED SAMPLE** | | | **HORTEGA POPULATION** | | | **SEGOVIA POPULATION** | | |
| --- | --- | --- | --- | --- | --- | --- | --- | --- | --- | --- |
| CHR | SNP | N_MISS | N_GENO | CALL_RATE | N_MISS | N_GENO | CALL_RATE | N_MISS | N_GENO | CALL_RATE |
| 1 | *c.-345 C>T* | 15 | 2022 | 99.25 | 3 | 1217 | 99.75 | 12 | 805 | 98.51 |
| 1 | *rs2271072* | 15 | 2022 | 99.25 | 2 | 1217 | 99.83 | 13 | 805 | 98.38 |
| 1 | *rs12401792* | 8 | 2022 | 99.60 | 0 | 1217 | 100 | 8 | 805 | 99.00 |
| 2 | *rs2197076* | 6 | 2022 | 99.70 | 0 | 1217 | 100 | 6 | 805 | 99.25 |
| 2 | *rs2241883* | 6 | 2022 | 99.70 | 2 | 1217 | 99.83 | 4 | 805 | 99.50 |
| 2 | *rs2970901* | 189 | 2022 | 90.65 | 83 | 1217 | 93.18 |  |  |  |
| 4 | *rs1511025* | 16 | 2022 | 99.20 | 4 | 1217 | 99.67 | 12 | 805 | 98.51 |
| 4 | *rs6857641* | 7 | 2022 | 99.65 | 1 | 1217 | 99.91 | 6 | 805 | 99.25 |
| 4 | *rs2282688* | 14 | 2022 | 99.30 | 9 | 1217 | 99.26 | 5 | 805 | 99.37 |
| 4 | *rs10034579* | 11 | 2022 | 99.45 | 11 | 1217 | 99.09 | 0 | 805 | 100 |
| 8 | *rs8192688* | 56 | 2022 | 97.23 | 50 | 1217 | 95.89 | 6 | 805 | 99.25 |
| 8 | *rs16909225* | 5 | 2022 | 99.75 | 3 | 1217 | 99.75 | 2 | 805 | 99.75 |
| 4 | *rs4834770** |  |  |  | 11 | 1217 | 99.09 | 0 | 805 | 100 |
| 1 | *rs2279885* |  |  |  | 23 | 1217 | 98.11 |  |  |  |
| 4 | *rs1799883* |  |  |  |  |  |  | 68 | 805 | 91.55 |

*SNP: SNP identifier; CHR: Chromosome number; N_MISS: Number of individuals missing this SNP; N_GENO: Number of non-obligatory missing genotypes; CALL_RATE: percentage of individuals with no missing genotypes for each SNP. * rs4834770 was excluded because was not in HWE in controls.*
