# Supplementary material for: Common Variants of the Liver Fatty Acid Binding Protein Gene Influence the Risk of Type 2 Diabetes and Insulin Resistance in Spanish Population
Source: PLoS One. 2012 Mar 2;7(3):e31853. doi: 10.1371/journal.pone.0031853 (PMC3292554; doi:10.1371/journal.pone.0031853)
Supplement: Table S2 — Association among genotypes of the rs2197076 and diabetes related traits under an additive inheritance genetic model. (DOCX) [file pone.0031853.s002.docx]

| **QUALITATIVE TRAITS*** | **HORTEGA** | | | **SEGOVIA** | | | **POOLED** | | |
| --- | --- | --- | --- | --- | --- | --- | --- | --- | --- |
|  | **OR** | **CI** | **p-value** | **OR** | **CI** | **p-value** | **OR** | **CI** | **p-value** |
| OBESITY^1^ | 0.82 | 0.60-1.15 | 0.29 | 1.06 | 0.78-1.45 | 0.69 | 0.94 | 0.75-1.17 | 0.59 |
| ABDOMINAL OBESITY^2^ | 0.83 | 0.65-1.06 | 0.14 | 0.93 | 0.70-1.24 | 0.65 | 0.86 | 0.72-1.03 | 0.12 |
| HYPERTRIGLYCERIDEMIA^3^ | 1.10 | 0.88-1.37 | 0.39 | **1.46** | **1.01-2.09** | **0.04** | **1.19** | **1.00-1.43** | **0.04** |
| HYPERTENSION^4^ | 0.95 | 0.76-1.18 | 0.65 | 1.09 | 0.83-1.42 | 0.52 | 1.00 | 0.84-1.18 | 0.97 |
| HYPOCHOLESTEROLEMIA HDL^5^ | 0.93 | 0.71-1.22 | 0.63 | 1.18 | 0.80-1.74 | 0.39 | 1.01 | 0.81-1.27 | 0.87 |
| HYPERCHOLESTEROLEMIA LDL ^6^ | 1.07 | 0.81-1.41 | 0.62 | **1.42** | **1.07-1.87** | **0.01** | 1.16 | 0.96-1.38 | 0.10 |
|  | | | | | | | | | |
| **QUANTITATIVE TRAITS**** | **HORTEGA** | | | **SEGOVIA** | | | **POOLED** | | |
|  | **BETA** | **CI** | **p-value** | **BETA** | **CI** | **p-value** | **BETA** | **CI** | **p-value** |
| TRIGLYCERIDES (mg/dl) | -2.41 | -13.5 – 8.74 | 0.67 | 3.64 | -6.5 – 13.8 | 0.48 | 1.35 | -7.05 – 9.75 | 0.75 |
| HDL CHOLESTEROL (mg/dl) | -0.01 | -1.35 – 1.33 | 0.98 | 0.19 | -2.0 – 2.42 | 0.86 | -0.04 | -1.24 – 1.16 | 0.94 |
| LDL CHOLESTEROL (mg/dl) | 0.32 | -3.30 – 3.95 | 0.86 | 1.18 | -3.6 - 6.01 | 0.63 | 0.14 | -2.91 – 3.19 | 0.92 |
| SBP (mmHg) | -0.24 | -2.49 – 2.0 | 0.83 | 0.86 | -1.52 –3.25 | 0.47 | 0.31 | -1.35 – 1.98 | 0.71 |
| DBP (mmHg) | -0.47 | -1.59 – 0.65 | 0.41 | 0.73 | -0.59 -2.06 | 0.27 | 0.02 | -0.83 – 0.88 | 0.96 |

HDL cholesterol: high density lipoprotein cholesterol; LDL cholesterol: low density lipoprotein cholesterol; SBP: systolic blood pressure; DBP: diastolic blood pressure;^*^ Adjusted by age, gender and BMI except obesity that was adjusted by age and gender; ^1^BMI≥30 kg/m^2^; ^2^ Waist circumference >102 in males and >88 in females; ^3^ Triglycerides levels equal or higher than150 mg/dl;^4^ Use or antihypertensive drugs or blood pressure over 140/90; ^5^ HDL cholesterol lower than 40 mg/dl; ^6^ LDL cholesterol higher than 160 mg/dl; ** Adjusted by age, sex, BMI and lipid treatment for triglycerides, HDL and LDL cholesterol and by age, sex, BMI and antihypertensive treatment for SBP and DBP. The most relevant results are shown in bold type.
