# Supplementary material for: Common Variants of the Liver Fatty Acid Binding Protein Gene Influence the Risk of Type 2 Diabetes and Insulin Resistance in Spanish Population
Source: PLoS One. 2012 Mar 2;7(3):e31853. doi: 10.1371/journal.pone.0031853 (PMC3292554; doi:10.1371/journal.pone.0031853)
Supplement: Table S3 — Association among polymorphisms of the FABP2 gene and hypertriglyceridemia under an additive inheritance genetic model. (DOCX) [file pone.0031853.s003.docx]

| **VARIANT** | **HORTEGA** | | | **SEGOVIA** | | | **POOLED** | | |
| --- | --- | --- | --- | --- | --- | --- | --- | --- | --- |
|  | **OR** | **CI** | **p-value** | **OR** | **CI** | **p-value** | **OR** | **CI** | **p-value** |
| *rs10034579* | **0.84** | **0.71-0.99** | **0.04** | **0.63** | **0.47-0.84** | **0.002** | **0.77** | **0.68-0.89** | **0.0002** |
| *rs6857641* | 0.84 | 0.71-1.0 | 0.05 | **0.62** | **0.46-0.84** | **0.002** | **0.77** | **0.67-0.88** | **0.0001** |
| *rs2282688* | 0.84 | 0.71-1.08 | 0.05 | **0.62** | **0.46-0.83** | **0.001** | **0.77** | **0.67-0.88** | **0.0001** |
| *rs4834770* | 0.92 | 0.78-1.0 | 0.3 | **0.63** | **0.46-0.85** | **0.002** |  |  |  |
| *rs1511025* | 0.94 | 0.79-1.13 | 0.5 | **1.44** | **1.06-1.9** | **0.01** | 1.08 | 0.93-1.25 | 0.26 |
