# Supplementary material for: Common Variants of the Liver Fatty Acid Binding Protein Gene Influence the Risk of Type 2 Diabetes and Insulin Resistance in Spanish Population
Source: PLoS One. 2012 Mar 2;7(3):e31853. doi: 10.1371/journal.pone.0031853 (PMC3292554; doi:10.1371/journal.pone.0031853)
Supplement: Table S4 — Alleles and genotypes frequencies for the analyzed polymorphism in the pooled sample, Hortega and Segovia population separated by type 2 diabetes status. (DOCX) [file pone.0031853.s004.docx]

|  | POOLED POPULATION | | | HORTEGA POPULATION | | | SEGOVIA POPULATION | | |
| --- | --- | --- | --- | --- | --- | --- | --- | --- | --- |
|  | ALL* | NO TYPE 2 DIABETES* | TYPE 2 DIABETES* | ALL* | NO TYPE 2 DIABETES* | TYPE 2 DIABETES* | ALL* | NO TYPE 2 DIABETES* | TYPE 2 DIABETES* |
| rs2197076 | G:3335(82.7) A:697(17.3) AA: 58(2.9) AG: 581(28.8) GG:1,377(68.3) Total:2,016(100) | G:3004(83.4) A:598(16.6) AA:52(2.9) AG:494(27.4) GG:1,255(69.7 Total:1,801(100) | G:261(75.0) A:87(25.0) AA:6(3.4) AG:75(43.1) GG:93(53.4) Total:174(100) | G:2000(82.2) A:434(17.8) AA:34(2.8) AG:366(30.1) GG:817(67.1) Total:1,217(100) | G:1857(82.8) A:387(17.2) AA:31(2.8) AG:325(29.0) GG:766(68.3) Total:1,122(100) | G:143(75.3) A:47(24.7) AA:3(3.2) AG:41(43.2) GG:51(53.7) Total:95(100) | G:1335(83.6) A:263(16.4) AA:24(3.0) AG:215(26.9) GG:560(70.1) Total:799(100) | G:1147(84.5) A:211(15.5) AA:21(3.1) AG:169(24.9) GG:489(72.0) Total:679(100) | G:118(74.7) A:40(25.3) AA:3.0( 3.8) AG:34(43.0) GG:42(53.2) Total:79(100) |
| rs224*1*883 | T:2619(65.0) C:1413(35.0) CC: 247(12.3) CT:919(45.6) TT:850(42.2) Total:2,016(100) | T:2349(65.2) C:1255(34.8) CC:217(12.0) CT:821(45.6) TT:764(42.4) Total:1,802(100) | T:223(64.1) C:125(35.9) CC:24(13.8) CT:77(44.3) TT:73(42.0) Total:174(100) | T:1595(65.7) C:835(34.3) CC:150(12.3) CT:535(44.0) TT:530(43.6) Total:1,215(100) | T:1468(65.6) C:772(34.4) CC:136(12.1) CT:500(44.6) TT:484(43.2) Total:1,120(100) | T:127(66.9) C:63(33.1) CC:14(14.7) CT:35(36.8) TT:46(48.4) Total:95(100) | T:1024(64.0) C:578(36.0) CC:97(12.1) C:T384(47.9) TT:320(40.0) Total:801(100) | T:881(64.6) C:483(35.4) CC:81(11.9) CT:321(47.1) TT:280(41.1) Total:682(100) | T:96(60.8) C:62(39.2) CC:10(12.7) CT:42(53.2) TT:27(34.2) Total:79(100) |
| rs2970901 | G:2257(61.6) T:1409(38.4) GG:692(37.8) GT:873(47.6) TT:268(14.6) Total:1,833(100) | G:2020(61.7) T:1258(38.3) GG:623(38.0) GT:774(47.2) TT:242(14.8) Total:1,639(100) | G:192(61.6) T:120(38.4) GG:56(35.9) GT:80(51.3) TT:20(12.8) Total:156(100) | G:1404(62.0) T:864(38.0) GG:435(38.4) GT:534(47.1) TT:165(14.6) Total:1,134(100) | G:1292(61.6) T:806(38.4) GG:399(38.0) GT:494(47.1) TT:156(14.9) Total:1,049(100) | G:112(65.9) T:58(34.1) GG:36(42.4) GT:40(47.1) TT:9(10.6) Total:85(100) | G:853(61.1) T:545(38.9) GG:257(36.8) GT:339(48.5) TT:103(14.7) Total:699(100) | G:728(61.7) T:452(38.3) GG:224(38.0) GT:280(47.5) TT:86(14.6) Total:590(100) | G:80(56.4) T:62(43.6) GG:20(28.2) GT:40(56.3) TT:11(15.5) Total:71(100) |
| rs1511025 | T:2787(69.5) C:1225(30.5) CC:196(9.8) CT:833(41.5) TT:977(48.7) Total:2,006(100) | T:2486(69.4) C:1098(30.6) CC:179(10.0) CT:740(41.3) TT:873(48.7) Total:1,792(100) | T:246(71.1) C:100(28.9) CC:13(7.5) CT:74(42.8) TT:86(49.7) Total:173(100) | T:1670(68.9) C:756(35.1) CC:117(9.6) CT:522(43.0) TT:574(47.3) Total:1,213(100) | T:1535(68.6) C:703(31.4) CC:110(9.8) CT:483(43.2) TT:526(47.0) Total:1,119(100) | T:135(71.8) C:53(28.2) CC:7(7.4) CT:39(41.5) TT:48(51.1) Total:94(100) | T:1117(70.5) C:469(29.5) CC:79(10.0) CT:311(39.2) TT:403(50.8) Total:793(100) | T:951(70.7) C:395(29.3) CC:69(10.3) CT:257(38.2) TT:347(51.6) Total:673(100) | T:111(70.3) C:47(29.7) CC:6(7.6) CT:35(44.3) TT:38(48.1) Total:79(100) |
| rs4834770* | G:2184(54.3) A:1838(45.7) AA:460(22.9) AG:918(45.6) GG:633(31.5) Total:2,011(100) | G:1954(54.4) A:1638(45.6) AA:411(22.9) AG:816(45.4) GG:569(31.7) Total:1,796(100) | G:178(51.2) A:170(48.8) AA:45(25.9) AG:80(46.0) GG:49(28.2) Total:174(100) | G:1283(53.2) A:1129(46.8) AA:293(24.3) AG:543(45.0) GG:370(30.7) Total:1,206(100) | G:1200(54.0) A:1022(46.0) AA:259(23.3) AG:504(45.4) GG:348(31.3) Total:1,111(100) | G:83(43.7) A:107(56.3) AA:34(35.8) AG:39(41.1) GG:22(23.2) Total:95(100) | G:901(56.0) A:709(44.0) AA:167(20.7) AG:375(46.6) GG:263.0(32.7) Total:805(100) | G:754(55.1) A:616(44.9) AA:152(22.2) AG:312(45.5) GG:221(32.3) Total:685(100) | G:95(60.2) A:63(39.8) AA:11(13.9) AG:41(51.9) GG:27(34.2) Total:79(100) |
| rs6857641 | C:2333(57.9) T:1697(42.1) CC:684(33.9) CT:965(47.9) TT:366(18.2) Total:2,015(100) | C:2083(57.9) T:1517(42.1) CC:612(34.0) CT:859(47.7) TT:329(18.3) Total:1,800(10) | C:198(56.9) T:150(43.1) CC:57(32.8) CT:84(48.3) TT:33(19.0) Total:174(100) | C:1437(59.1) T:995(40.9) CC:421(34.6) CT:595(48.9) TT:200(16.4) Total:1,216(100) | C:1334(59.5) T:908(40.5) CC:391(34.9) CT:552(49.2) TT:178(15.9) Total:1,121(100) | C:103(54.3) T:87(45.7) CC:30(31.6) CT:43(45.3) TT:22(23.2) Total:95(100) | C:896(56.1) T:702(43.9) CC:263(32.9) CT:370(46.3) TT:166(20.8) Total:799(100) | C:749(55.2) T:609(44.8) CC:221(32.5) CT:307(45.2) TT:151(22.2) Total:679(100) | C:95(60.2) T:63(39.8) CC:27(34.2) CT:41(51.9) TT:11(13.9) Total:79(100) |
| rs2282688 | G:2326(57.9) A:1690(42.1) AA:364(18.1) AG:962(47.9) GG:682(34.0) Total:2,008(100) | G:2077(57.9) A:1513(42.1) AA:327(18.2) AG:859(47.9) GG:609(33.9) Total:1,795(100) | G:197(57.3) A:147(42.7) AA:33(19.2) AG:81(47.1) GG:58(33.7) Total:172(100) | G:1430(59.2) A:986(40.8) AA:198(16.4) AG:590(48.8) GG:420(34.8) Total:1,208(100) | G:1327(59.6) A:901(40.4) AA:176(15.8) AG:549(49.3) GG:389(34.9) Total:1,114(100) | G:103(54.8) A:85(45.2) AA:22(23.4) AG:41(43.6) GG:31(33.0) Total:94(100) | G:896(56.0) A:704(44.0) AA:166(20.8) AG372(46.5) GG:262(32.8) Total:800(100) | G:750(55.1) A:612(44.9) AA:151(22.2) AG:310(45.5) GG:220(32.3) Total:681(100) | G:94(60.3) A:622(39.7) AA:11(14.1) AG:40(51.3) GG:27(34.6) Total:78(100) |
| rs10034579 | C:2312(57.5) A:1710(42.5) AA:379(18.8) AC:952(47.3) CC:680(33.8) Total:2,011(100) | C:2066(57.5) A:1530(42.5) AA:341(19.0) AC:848(47.2) CC:609(33.9) Total:1,798(100) | C:194(56.4) A:150(43.6) AA:34(19.8) AC:82(47.7) CC:56(32.6) Total:172(100) | C:1411(58.5) A:1001(41.5) AA:212(17.6) AC:577(47.8) CC:417(34.6) Total:1,206(100) | C:1312(59.0) A:914(41.0) AA:189(17.0) AC:536(48.2) CC:388(34.9) Total:1,113(100) | C:99(53.3) A:87(46.7) AA:23(24.7) AC:41(44.1) CC:29(31.2) Total:93(100) | C:901(56.0) A:709(44.0) AA:167(20.7) AC:375(46.6) CC:263(32.7) Total:805(100) | C:754(55.1) A:616(44.9) AA:152(22.2) AC:312(45.5) CC:221(32.3) Total:685(100) | C:95(60.2) A:63(39.8) AA:11(13.9) AC:41(51.9) CC:27(34.2) Total:79(100) |
| rs2271072 | C:2554(63.3) G:1460(36.7) CC:797(39.7) CG:960(47.8) GG:250(12.5) Total:2,007(100) | C:2283(63.3) G:1307(36.4) CC:713(39.7) CG:857(47.7) GG:225(12.5) Total:1,795(100) | C:212(62.0) G:130(38.0) CC:62(36.3) CG:88(51.5) GG:21(12.3) Total:171(100) | C:1530(63.0) G:900(37.0) CC:469(38.6) CG:592(48.7) GG:154(12.7) Total:1,215(100) | C:1414(63.1) G:828(36.9) CC:436(38.9) CG:542(48.3) GG:143(12.8) Total:1,121(100) | C:116(61.7) G:72(38.3) CC:33(35.1) CG:50(53.2) GG:11(11.7) Total:94(100) | C:1024(64.7) G:560(35.3) CC:328(41.4) CG:368(46.5) GG:96(12.1) Total:792(100) | C:869(64.5) G:479(35.5) CC:277(41.1) CG:315(46.7) GG:82(12.2) Total:674(100) | C:96(62.4) G:58(37.6) CC:29(37.7) CG:38(49.4) GG:10(13.0) Total:77(100) |
| rs2279885 | G:2316(98.5) C:36(1.50) CG:36(3.0) GG:1,158(97.0) Total:1,194(100) | G:2169(98.5) C:33(1.5) CG:33(3.0) GG:1,068(97.0) Total:1,101(100) | G:183(98.4) C:3(1.6) CG:3(3.2) GG:90(96.8) Total:93(100) | G:2352(98.5) C:36(1.5) CG:36(3.0) GG:1,158(97.0) Total:1,194(100) | G:2169(98.5) C:33(1.5) CG:33(3.0) GG:1,068(97.0) Total:1,101(100) | G:183(98.4) C:3(1.6) CG:3(3.2) GG:90(96.8) Total:93(100) |  |  |  |
| c.-345 C>T | C:3229(80.5) T:785(19.5) CC:1,298(64.7) TC:633(31.5) TT:76(3.8) Total:2,007(100) | C:2885(80.4) T:703(19.6) CC:1,159(64.6) TC:567(31.6) TT:68(3.8) Total:1,794(100) | C:276(79.8) T:70(20.2) CC:109(63.0) TC:58(33.5) TT:6(3.5) Total:173(100) | C:1925(79.3) T:503(20.7) CC:774(63.8) TC:395(32.5) TT:45(3.7) Total:1,214(100) | C:1790(80.0) T:448(20.0) CC:713(63.7) TC:364(32.5) TT:42(3.8) Total:1,119(100) | C:153(80.6) T:37(19.4) CC:61(64.2) TC:31(32.6) TT:3(3.2) Total:95(100) | C:1286(81.1) T:300(18.9) CC:524(66.1) TC:238(30.0) T:T31(3.9) Total:793(100) | C:1095(81.2) T:255(18.8) CC:446(66.1) TC:203(30.1) TT:26(3.9) Total:675(100) | C:123(78.9) T:33(21.1) CC:48(61.5) TC:27(34.6) TT:3(3.8) Total:78(100) |
| rs12401792 | G:3098(77.0) T:930(23.0) GG:1,190(59.1) GT:718(35.7) TT:106(5.3) Total:2,014(100) | G:2780(77.2) T:824(22.8) GG:1,069(59.3) GT:642(35.6) TT:91(5.0) Total:1,802(100) | G:252(73.7) T:90(26.3) GG:93(54.4) GT:66(38.6) TT:12(7.0) Total:171(100) | G:1850(76.0) T:584(24.0) GG:698(57.4) GT:454(37.3) TT:65(5.3) Total:1,217(100) | G:1712(76.3) T:532(23.7) GG:648(57.8) GT:416(37.1) TT:58(5.2) Total:1,122(100) | G:138(72.7) T:52(27.3) GG:50(52.6) GT:38(40.0) TT:7(7.4) Total:95(100) | G:1248(78.3) T:346(21.7) GG:492(61.7) GT:264(33.1) TT:41(5.1) Total:797(100) | G:1068(78.6) T:292(21.4) GG:421(61.9) GT:226(33.2) TT:33(4.9) Total:680(100) | G:114(75.0) T:38(25.0) GG:43(56.6) GT:28(36.8) TT:5(6.6) Total:76(100) |
| rs8192688 | C:3384(86.1) T:548(13.9) CC:1,466(74.6) CT:452(23.0) TT:48(2.4) Total:1,966(100) | C:3024(86.0) T:492(14.0) CC:1,308(74.4) CT:408(23.2) TT:42(2.4) Total:1,758(100) | C:284(85.0) T:50(15.0) CC:123(73.7) CT:38(22.8) TT:6(3.6) Total:167(100) | C:1992(85.4) T:342(14.6) CC:862(73.9) CT:268(23.0) TT:37(3.2) Total:1,167(100) | C:1701(84.5) T:313(15.5) CC:796(73.9) CT:249(23.1) TT:32(3.0) Total:1,077(100) | C:151(83.9) T:29(16.1) CC:66(73.3) CT:19(21.1) TT:5(5.6) Total:90(100) | C:1392(87.1) T:206(12.9) CC:604(75.6) CT:184(23.0) TT:11(1.4) Total:799(100) | C:1183(86.9) T:179(13.1) CC:512(75.2) CT:159(23.3) TT:10(1.5) Total:681(100) | C:133(86.4) T:21(13.6) CC:57(74.0) CT:19(24.7) TT:1(1.3) Total:77(100) |
| rs16909225 | A:3465(85.9) G:569(14.1) AA:1,485(73.6) AG:495(24.5) GG:37(1.8) Total:2,017(100) | A:3091(85.8) G:513(14.2) AA:1,324(73.5) AG:443(24.6) GG:35(1.9) Total:1,802(100) | A:298(85.7) G:50(14.3) AA:126(72.4) AG:46(26.4) GG:2(1.1) Total:174(100) | A:2084(85.9) G:344(14.1) AA:895(73.7) AG:294(24.2) GG:25(2.1) Total:1,214(100) | A:1921(85.9) G:317(14.1) AA:826(73.8) AG:269(24.0) GG:24(2.1) Total:1,119(100) | A:163(85.8) G:27(14.2) AA:69(72.6) AG:25(26.3) GG:1(1.1) Total:95(100) | A:1381(86.0) G:225(14.0) AA:590(73.5) AG:201(25) GG:12(1.5) Total:803(100) | A:1170(85.7) G:196(14.3) AA:498(72.9) AG:174(25.5) GG:11(1.6) Total:683(100) | A:135(85.5) G:23(14.5) AA:57(72.2) AG:21(26.6) GG:1(1.3) Total:79(100) |
| rs1799883 | G:2534(74.2) A:916(25.8) AA:130(7.3) GA:656(37.1) GG:984(55.6) Total:1,770(100) | G:2320(73.7) A:828(26.3) AA:119(7.6) GA:590(37.5) GG:865(55.0) Total:1,574(100) | G:238(75.4) A:78(24.6) AA:10(6.3) GA:58(36.7) GG:90(57.0) Total:158(100) | G:1533(74.2) A:533(25.8) AA:71(6.9) GA:391(37.9) GG:571(55.3) Total:1,033(100) | G:1411(74.6) A:481(25.4) AA:62(6.6) GA:357(37.7) GG:527(55.7) Total:946(100) | G:122(70.2) A:52(29.8) AA:9(10.3) GA:34(39.1) GG:44(50.6) Total:87(100) | G:1091(74.1) A:383(25.9) AA:59(8.01) GA:265(35.9) GG:413(56.0) Total:737(100) | G:909(72.4) A:347(27.6) AA:57(9.1) GA:233(37.1) GG:338(53.8) Total:628(100) | G:116(81.7) A:26(18.3) AA:1(1.4) GA:24(33.8) GG:46(64.8) Total:71(100) |

**Cells are expressed as total number (percentage). The results for the main associated SNP have been remarked. * rs4834770 was excluded because was not in HWE in controls.*
